# Talking on the Phone While Driving: A Literature Review on Driving Simulator Studies

**DOI:** 10.3390/ijerph191710554

**Published:** 2022-08-24

**Authors:** Răzvan Gabriel Boboc, Gheorghe Daniel Voinea, Ioana-Diana Buzdugan, Csaba Antonya

**Affiliations:** Department of Automotive and Transport Engineering, Transilvania University of Brașov, 29 Eroilor Blvd., 500036 Brasov, Romania

**Keywords:** mobile phone, distraction, driving simulator, scoping review

## Abstract

Distracted driving is a growing concern around the world and has been the focus of many naturalistic and simulator-based studies. Driving simulators provide excellent practical and theoretical help in studying the driving process, and considerable efforts have been made to prove their validity. This research aimed to review relevant simulator-based studies focused on investigating the effects of the talking-on-the-phone-while-driving distraction on drivers’ behavior. This work is a scoping review which followed the Preferred Reporting Items for Systematic Reviews and Meta-Analysis Extension for Scoping Reviews (PRISMA-ScR) guidelines. The search was performed on five databases, covering twenty years of research results. It was focused on finding answers to three research questions that could offer an overview of the main sources of distraction, the research infrastructure, and the measures that were used to analyze and predict the effects of distractions. A number of 4332 studies were identified in the database search, from which 83 were included in the review. The main findings revealed that TPWD distraction negatively affects driving performance, exposing drivers to dangerous traffic situations. Moreover, there is a general understanding that the driver’s cognitive, manual, visual, and auditory resources are all involved, to a certain degree, when executing a secondary task while driving.

## 1. Introduction

Distracted driving is a growing concern around the world. According to the National Highway Traffic Safety Administration (NHTSA), there were 36,096 people killed in car traffic accidents on U.S. roadways in 2019, and 8.7 percent of all fatalities were caused by distracting factors [1]. In Europe, even though the number of road deaths in 2020 was de-clined compared to 2019, an estimated 18,800 people were killed in a road crash last year [2]. Romania recorded the highest rate in the European Union, with 85 road fatalities per million inhabitants [3]. Along with speeding, driving under the influence of alcohol, and non-use of seatbelts, distracted driving is one of the main causes of death in traffic acci-dents [4]. Although there are other causes, human factors still play an essential role in to-day’s collisions, primarily due to “multitasking” while driving, which affects driving performance [5].

The present study, designed as a scoping review, joins the efforts of other authors who have conducted review articles related to the study of various topics in driving sim-ulators, such as the impact of cannabis on the driving performance of novice drivers [6], road geometric design effects on driver behavior [7], awareness of sleepiness while driv-ing [8], the influence of alcohol on driving behavior [9], sleepiness detection [10], drowsi-ness effect [11], fitness to drive in sleepy individuals [12], relationship between impact speed and the probability of pedestrian fatality during a vehicle–pedestrian crash [13], driving simulator validation [14], driving simulation technology and applications [15], and driving performance parameters critical for distracted driving research [16]. A systematic search of the literature was performed to highlight the studies conducted in driving simulators that are focused on the detection of distractions caused by the use of mobile phones and what is the knowledge that emerges from these studies. The following research questions (RQ) were defined: 

RQ1: What types of distractions are introduced when talking on the phone while driving?

RQ2: What types of hardware devices were used during experiments to analyze the driver’s behavior?

RQ3: What measures were used to predict and analyze distractions?

The impact of mobile phone distraction on driving performance was analyzed in a previous study [17]. However, this article tries to add new elements and summarize up-to-date knowledge regarding simulator-based investigations of talking-while-driving distractions. Section 1 and Section 2 present the context and the need for the current work. Section 3 describes the research methodology and the research questions (RQ). Section 4 presents the results, with a focus on answering to the three RQs. An important contribution is also the overview of the measures used to quantify distracted driving in a simulator which may help scholars when designing their studies. Section 5 presents the main findings and proposed recommendations for future research. Lastly, Section 6 presents the conclusions and the limitations of the work.

## 2. Background

Distracted driving refers to any activity that takes the driver’s attention away from driving. Eating, drinking, or adjusting vehicle controls are considered distraction sources [18]. These causes of distraction, alongside smoking, reaching for an object, reading, conversing, or grooming, can be referred to as in-vehicle distractions, as they are caused by sources inside the vehicle [19]. External distractions (outside-of-vehicle) can refer to unimportant events or objects outside the car that the driver is focusing on [20], such as billboards, work zones, crash scenes, police cars [21], or, in other words, the rubbernecking at on-road events [22]. Recent studies showed that interaction with a passenger, entertainment, mobile devices, and external scenes are the most common distractions for drivers of different age groups [23,24].

On the other hand, distractions can be classified regarding the driver: visual, manual (physical), cognitive, or auditive [25]. Some distracting activities can include individual elements or combinations of the four types [26]. Talking on the phone and texting are secondary activities that require all of the driver’s resources [27], and several studies have concluded that this activity is one with a high risk of crashes [28,29,30]. Moreover, other findings have revealed that there is a significantly increased risk for a driver to be involved in a car crash when exposed to activities that require looking away from the traffic scene (visual distraction), taking one or both hands off the wheel (manual distraction) [31], or mentally focus on something different than the driving task (cognitive and acoustic distraction) [32]. The additional resources needed when interacting with technological devices inside the car or with other passengers compete with the driving task [22]. Thus, distractions reduce the driving capability, leading to a degraded driving performance [33].

Research regarding the effects of using a mobile phone while driving requires more attention from scholars and legislators, as the number of mobile phone users worldwide has seen exponential growth in the last decade. Globally, there are 5.27 billion unique mobile phones today, representing 66.9% of the total population [34]. The ubiquity of smartphones is undoubted, affecting the driving process, and represents a significant public health threat [35,36]. Talking on the phone while driving (TPWD) can have a wide range of unfavorable consequences [37,38], and contribute to the number of distraction-affected crashes [39]. Whenever a notification is received, the persistent need to check the mobile phone while driving is very present, especially among young people. Even if the use of a mobile phone while driving is illegal in most countries of the world [40], younger adults perceive the risks associated with using mobile phones as “a natural and acceptable component of driving” [41]. This spread of mobile phone activities and the fact that smartphone users are unaware of their dependence [42] involves adopting some countermeasures such as public education messages [43] to reduce the risks associated with TPWD.

Moreover, mobile phone addiction has been shown to affect social relationships and academic achievements [44], learning [45], and even just looking at the phone’s display influences face-to-face communication [46]. However, it becomes an additional problem when it overlaps with the activity of driving a car. According to the World Health Organization (WHO), the risk of drivers who use mobile phones being involved in a crash, compared to those who do not use such devices, is about four times higher [47]. A study conducted in Italy (n = 774) revealed that 63.6% of drivers use their phones to initiate calls, 75.2% answer calls, 49.1% read messages, and 33.3% write them while driving [48]. Even when using a hands-free (HF) phone, studies based on experiments in real environments have shown that drivers are less focused on the traffic scene, thus filtering information from the driving environment [49]. 

Researchers have employed naturalistic and simulator-based studies to better understand the driver’s behavior while engaged in secondary activities. Driving simulators provide excellent practical and theoretical help in studying the driving process [50], and considerable efforts have been made to prove their validity [51]. Even if they are very attractive for research, providing safeness for the driver, the experimenter, and other road participants [52], ease of data collection, and control and efficiency [9], cost, and prediction of real-world performance [53], driving simulators do not lack shortcomings: the simplest ones can degrade the accuracy of self-displacement or inter-vehicular distance perception [54], but also the most sophisticated does not provide all the visual, vestibular, and proprioceptive interactions that occur when driving at certain speeds [55]. It is obvious that a high validity of the results is obtained when high-fidelity simulation systems are used [56]. Regarding mobile phone conversation distraction, some authors tried to identify possible explanations for the apparent inconsistency in findings between naturalistic driving studies and experimental/simulator studies [57]. 

The studies included in the review have targeted a large number of measures to evaluate the driver’s performance or behavior, therefore, making it relatively difficult to compare the findings between the studies. Although most papers have reached similar conclusions, some claim that the effects of phone conversations do not have a significant impact on driving performance [58]. This result could be explained by the limited number of analyzed measures, which were the mean velocity and the standard deviation of lateral and longitudinal control. Moreover, the fidelity of the driving simulator can also affect the results. Low fidelity simulators have the advantage of avoiding the risk of motion sickness, with their fixed base and a smaller degree of immersion. Although they have well-documented results, the lack of a motion system and of a realistic driving environment can present serious limitations. The studies from this work have been classified according to the criteria presented in [59], which proposed four classes of driving simulators based on existing flight simulator classification standards.

## 3. Materials and Methods

The scoping review was based on the Preferred Reporting Items for Systematic Reviews and Meta-Analyses Extension for Scoping Reviews (PRISMA-ScR) [60]. Updated guidance on the methodology is provided in [61].

### 3.1. Protocol and Registrations

There was no other protocol than the following the descriptions presented below, which the authors conceived following the instructions of PRISMA-ScR statements.

### 3.2. Eligibility Criteria

The general inclusion criteria for the selected literature were the following: the paper had to be original research available in full text and published in a peer-reviewed journal in the English language; the paper had to be related to the use of mobile phones for talking, and to contain a study performed on car simulators. 

In order to ensure a high level of quality of the papers, we have included only journal articles, and the following items were excluded: short papers, literature reviews, magazines, theses, editorials, book chapters, conference papers, and non-academic publications. Additionally, articles not available in full text or irrelevant to the research were excluded. 

The papers that focused on evaluating phone use for activities other than talking (e.g., texting) were excluded. However, the article in which two or more tasks were investigated, including talking on the phone, was included in the analysis. Regarding the year of publication, no time limit was applied.

### 3.3. Information Sources

The search was performed on 4 February 2021 using the following electronic engines: I.S.I. Web of Knowledge, Scopus, Science Direct, SAGE Journals, and ProQuest. An updated search was conducted on 24 May 2021 using the same databases.

### 3.4. Search

**Figure 1:**
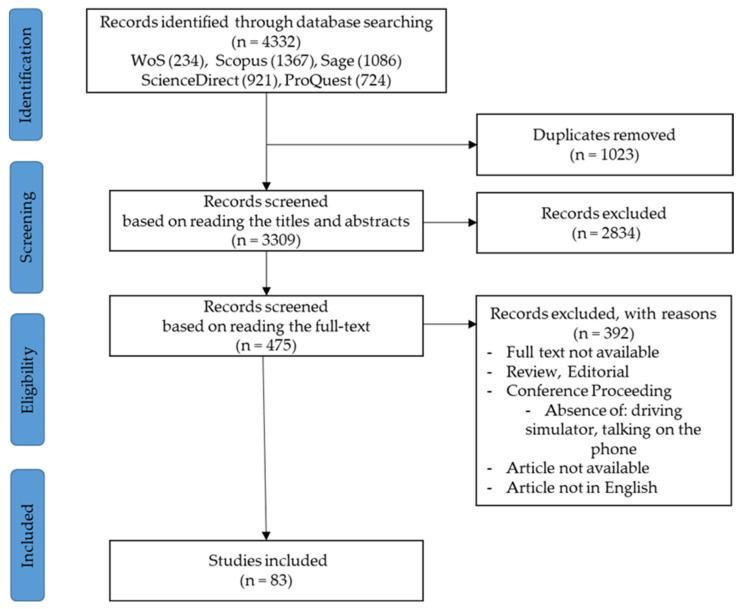
Study selection methodology following the PRISMA flow diagram.

The search strategy including combination of keywords “distraction”, “phone”, and “driving simulator”, and extrapolations of them: “distracted”, “disruptive”, “smartphone”, “mobile phone”, “cell phone”, “simulation”, and “simul*”. The search string applied for the Science Direct database was: (distracted OR disturbing) AND “driver behavior” AND (car OR vehicle OR automobile) AND (simulator OR “virtual environment” OR “simulated environment”). The flow diagram of the searching procedure is presented in Figure 1. The search included all the results containing mobile phones and driving simulators. In the screening phase, the papers unrelated to the talking task were removed. 

### 3.5. Selection of Source of Evidence

The literature extracted from the above-mentioned databases was processed using the tool EndNote (version 20, Clarivate, Philadelphia, PA, USA). In the first step, the duplicates were removed, and the remaining papers were screened on title and abstract. After removing the irrelevant articles, a full-text screening was performed, applying the eligibility criteria. The two authors conducted the screening independently, and the lead author intervened when there were doubts between the two.

### 3.6. Data Charting Process 

The authors developed a data charting process to identify relevant information to be included in the review. The author team reviewed the full text of eligible studies and identified data that could answer the proposed research questions. The data were charted using Microsoft Excel. Two authors performed data extraction (R.G.B. and G.D.V.), which was validated by the third (C.A.).

### 3.7. Data Items

The extracted information grouped into four categories is presented in Table 1. 

## 4. Results

The database search generated 4332 papers. After removing the duplicates, 3309 articles remain that were screened by the title and abstract. In this phase, 1023 articles were excluded, with 475 remaining. The full-text screening produced 185 results, but 43 were excluded for different reasons (see Figure 1). Finally, the papers addressing other types of phone use than talking were excluded, resulting 83 articles that were included in the final analysis. In addition to the discussion in the following sections, some characteristics of the papers are briefly presented in Table A1 from Appendix A.

### 4.1. Characteristics of Studies

**Figure 2:**
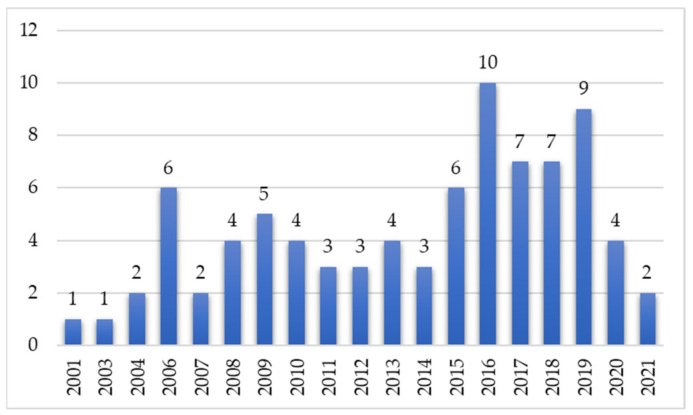
Distribution of papers by publication year.

The 83 studies that were considered for the review cover a range of twenty years (2001–2021). The number of references varies since 2001 from 1 to 10 (Figure 2) with an increasing evolution. Most published articles were in 2016 (n = 10). 

**Figure 3:**
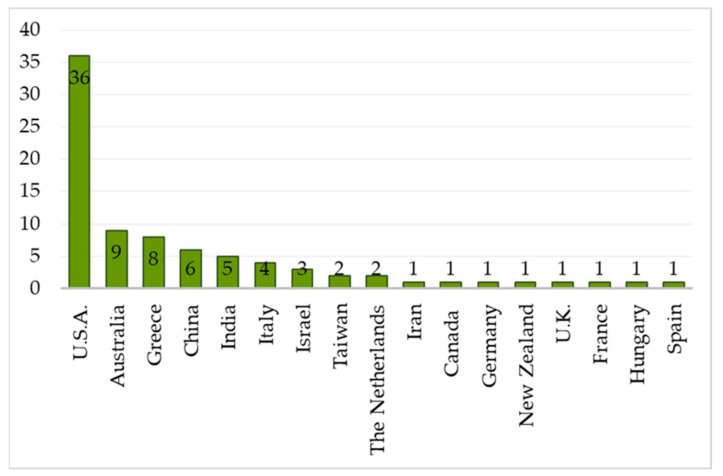
Distribution of papers by countries.

Most of the studies were developed in North America (n = 37), especially in the USA (n = 36) (Figure 3). The other studies were conducted in Europe (n = 19), Asia (n = 17), and Oceania (n = 10). In Europe, most publications are from Greece (n = 8) and Italy (n = 4). In Asia, most of the publications are from China (n = 6) and India (n = 5), and from Oceania, most studies have been developed in Australia (n = 9). 

Australian, American, and Greek research institutions dominate the total number of articles assessing TPWD distraction’s impact in virtual environments (Table 2). Most studies were developed at Queensland University of Technology (n = 8), followed by the University of Utah (n = 7), National University of Athens (n = 6), and Indian Institute of Technology (I.I.T.), Bombay (n = 4). 

In terms of the journal in which the studies were published, the screening phase resulted in articles published in the following journal sources: *Accidents Analysis and Prevention* (n = 21), *Transportation Research Part F: Traffic Psychology and Behaviour* (n = 11), *Transportation Research Record* (n = 9), and other journals with 5, 4, 2 publications, respectively, one publication (Table 2). In the table, only the institutions and the journals with more than one publication are reported.

**Figure 4:**
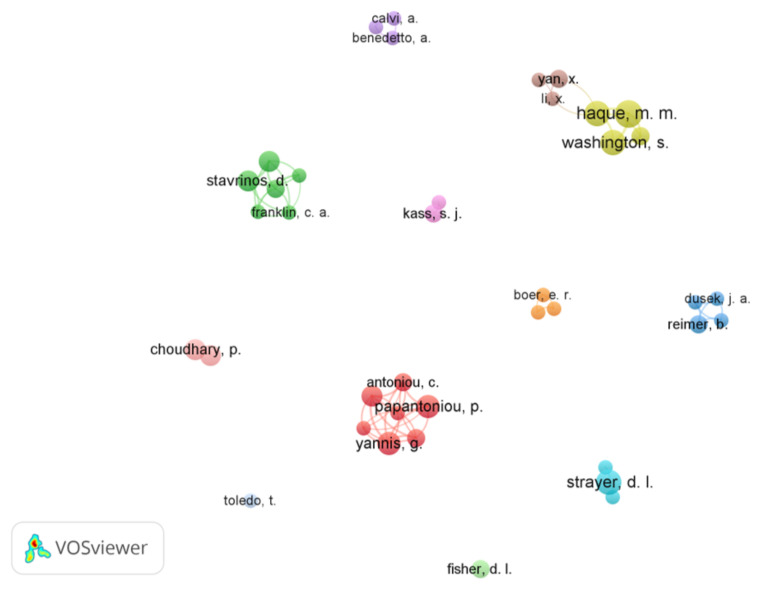
The network of co-authorship; node size indicates the number of papers (used VOSviewer 1.6.16, Leiden University, Leiden, The Netherlands, N = 182).

Authors affiliated with institutions in Australia, Greece, and the USA represent the majority of publications. The author–research network created in VOSViewer is presented in Figure 4. The minimum number of documents of an author was set to two to build a co-authored visual network map of 39 items from 252.

The selected studies included 4254 participants who participated in simulated driving experiments. The minimum number is 14 [62], and the maximum is 140 [63] participants per study. Some of the studies presented two experiments: [64,65,66,67], or split the sample in two or three: inexperienced drivers vs. experienced drivers [68,69,70], young vs. older participants [71,72,73], novice drivers and young adults [74], young-, middle-, old-age [75], experienced, novice, and older drivers [67], but the total number of participants was considered in this analysis. Other studies used a control group in their experiments: [63,76,77,78,79,80,81,82].

The participants’ age ranges from 16 to 77, with the remark that in 17 studies, the age range is not given. However, 68 studies revealed the mean age, and the unweighted mean age for all these studies is 31.26 years. In most cases, the standard deviation is included, and it is 4.23 in all studies that mention it. However, in 28 research studies, the standard deviation for the age distribution of the participants is not mentioned, and 15 studies do not report the mean age. Nevertheless, only four studies mention neither the age range, nor mean age, nor standard deviation.

In terms of health, all participants were considered to be in clinically good health, except for five studies which focused on: young adults with attention deficit hyperactivity disorder (ADHD) [83], teens with and without ADHD [81], drivers with Mild Cognitive Impairment [77], participants with Alzheimer’s disease, Parkinson’s disease, and Mild Cognitive Impairment [63], and participants with Glaucoma [79].

Regarding the driving experience of the participants, in 61 of the articles, this is not specified. In three of the articles, it is mentioned in the annual mileage, and in the rest of the studies, it is specified in the elapsed time since they obtained their driving license and varies between 3 months and 40.3 years. Of the total number of articles, 47% (n = 39) specify the average years of experience for the participants, and it is 12.6 across all these studies. Standard deviation was also mentioned in 26 references, having an average value of 4.39 for these studies. 

From the total number of references, 11 studies do not mention gender distribution. For the rest, 59.3% (n = 2197) of participants are male and 40.7% (n = 1506) are female. 

### 4.2. RQ1: What Types of Distractions Are Introduced When Talking on the Phone While Driving?

Studies based on driving simulators have shown that the driver’s performance can be compromised when performing secondary driving tasks [70]. Distraction can be achieved by removing the driver’s gaze (visual distraction) or mind (cognitive distraction) from the road for a certain period of time, which in some cases can prove to be fatal. The use of a mobile phone for talking was found to introduce cognitive demands [82,83].

To identify the sources of distraction, for each analyzed paper, we extracted the information regarding the most apparent component regarding the type of distraction. We categorized it into four categories, according to [84,85]: (V) visual, (Au) auditory, (M) manual (physical), and (C) cognitive distraction, in order to identify the sources of distraction that were used in the experiments. For example, visual distractions include: using in-vehicle devices [86], accessing and using smartphone applications while driving [87], and so on. Auditory distractions occur when drivers divert their attention from the road to other noises, such as the radio, the phone rings, and so on. Eating [26], drinking [25], and doing anything other than manipulating the steering wheel are examples of manual distractions. Lastly, cognitive distractions happen when the driver’s mind is preoccupied with anything else and cannot perceive what is crucial on the road. According to studies, all these distractions may be introduced by TPWD, and even if they last only a short duration, they may result in driving errors and even fatalities [88]. However, most tasks unrelated to the driving task involve combining these four modes [89].

**Figure 5:**
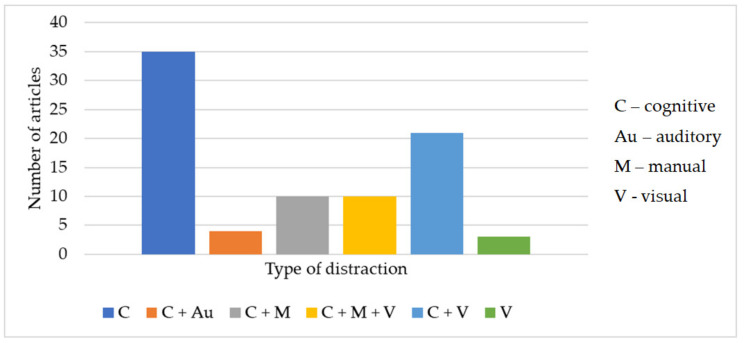
Distribution of papers by the source of distraction type.

The distribution of the papers by the source of distraction is presented in Figure 5. As can be seen, most articles (42.16% of the total number of papers, n = 35) considered the cognitive component when assessing the effects of secondary tasks while driving. As already mentioned, each secondary task contains one or more components, but we have classified them according to the study’s main objective and findings.

While some articles focused on the cognitive component, especially to find out how a hands-free phone conversation affects the driver’s performance [66,71,76,90,91,92,93], others considered two, three, or even four types of distractions. For example, cognitive and manual components were analyzed in [94,95,96], cognitive and visual components were presented in [25,97], and cognitive, visual, and manual attributes of the distraction were evaluated in [26,98] or [99]. As we have seen, only one article considered all four components of distraction: [100]. 

In various scenarios, the influence of the secondary task while driving was evaluated. From all the examined studies, two types of tasks were identified as predominant. In 20.48% of the total number of articles (n = 17), a car-following scenario was used, which requires following a lead vehicle and reacting to it [101]. In addition, in 60 studies (72.28% of studies), the first task was to freely drive along a path or route where one or more collisions happen. Good examples of such situations include: an animal suddenly appearing on the road [25,63,102,103], a pedestrian suddenly crossing the street [63,76,81,83,96,102,103,104,105,106,107,108,109,110,111,112,113,114], a cyclist entering the road [81,96,115], a parked car pulling out onto the road [104,109,110], a traffic signal intersection [76,109], and so on.

In addition to car-following and free driving situations, two articles included a dilemma zone situation—a situation that occurs near a traffic light intersection, in which the driver must decide to stop or continue when the light signal changes from green to yellow [116,117]. The other articles contain the following scenarios: 18 decision points at yellow onset [75], a roundabout [118], wayfinding [82], and following a moving target [65]. 

In their studies, some researchers have compared cell phone use with other activities, such as: talking to a passenger (12 studies: [62,63,77,91,102,103,119,120,121,122,123,124]), eating (2 studies: [26,88]), radio tuning (3 studies: [70,121,125]), radio listening [65], using navigation systems (3 studies: [52,70,89]), interaction with infotainment systems [98], adjusting climate controls [125], reading e-mails [126], drinking [25], and coin searching [97]. Three studies compare the use mobile phones while driving with drunk driving: [95,127,128]. 

**Figure 6:**
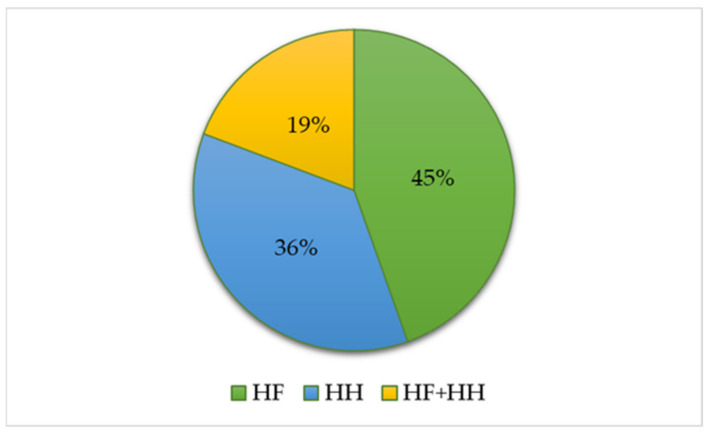
Distribution of papers by types of distraction task (HH—hand-held, HF—hands-free).

There were two categories for the distracting task: hand-held (HH)—holding the device in hand—or hands-free (HF)—performing the task without using hands to hold the device. The pie chart in Figure 6 shows the distribution of the articles according to these two sources of distraction. It can be observed that in 45% of the studies (n = 37), the phone conversation was performed using an HF device, in 30 articles, HH devices were used, and both HF and HH use was assessed in 16 studies. HF was preferred in 55% of the studies (n = 35), HH in 23% of the studies (n = 15), and both HH and HF in 22% of studies (n = 14). For PM, in 90% of the studies (n = 36), the HH device was used, while 7% of the studies used HF devices, and only in 3% of the studies were both HH and HF tasks evaluated. From the category of those who investigated both P.C. and P.F., 68% of them (n = 13) used HH devices, while 32% of them (n = 6) used HH and HF devices.

While some tasks involved real conversations and everyday topics, such as family, origin, accommodation, traveling [103], job or school commitments [58], daily driving habits, driving history, and personal information [82], in some studies, conversations involving a certain level of emotional involvement [129,130] or the use of the visuospatial ability [92,131] have been used. Tasks involving mathematical operations have been found in a large number of studies (n = 16), either with a simple level of difficulty, such as arithmetic problems [112,118,132], or with both simple and complex levels [105,133].

### 4.3. RQ2: What Types of Hardware Devices Were Used during Experiments to Analyze the Driver’s Behavior?

Regarding the hardware utilized in the research, there is a wide variety of tracking devices and driving simulators. Most studies share common characteristics in how the experiment is carried out, such as the fact that each experiment began with a justification of the aim of the research followed by a description of the tasks to be performed. After the participant gave their verbal or written agreement, they would undertake a training session to get accustomed to the driving simulator. The experiment included, in general, a baseline scenario that would then be compared with a distracted driving scenario. The distracting factor could come from a mobile phone, an in-vehicle device, or other mobile devices. Finally, participants were asked to complete a questionnaire to perform a subjective evaluation that could help to reveal new insights regarding the driver’s behavior under simulated conditions.

Regarding the type of simulators used to study different traffic situations in the examined studies, 75.9% of experiments (n = 63 studies) were performed in fixed-based simulators. Almost a quarter of the studies were conducted in driving simulators that allowed between 1 to 13 degrees of freedom (D.O.F.). The most advanced is the NADS-1 simulator used in [75,116,134]. It is located at the University of Iowa and provides 13 D.O.F. of motion, consisting of an entire car housed inside a dome assembled on the upper part of a turntable mounted on top of a hexapod [15]. Other simulators have only six D.O.F., such as the CARRS-Q Advanced Driving Simulator located at the Queensland University of Technology (Q.U.T.), Australia [55,93,99,107,112,118,135], a Nissan Maxima cab mounted on a hexapod motion-based located at the Center for Advanced Vehicular Systems, Mississippi State University, USA [136], and Ford’s VIRtual Test Track EXperiment [125], VS500M driving simulator [137]. Two experiments were performed in driving simulators with three D.O.F.: [98,127], two experiments were performed with two D.O.F. driving simulators: [76,138], and in three studies, the simulator had only one D.O.F.: the driving simulator located at Beijing Jiaotong University [101,132,133].

In some studies, custom-made driving simulators were used. These include a desktop computer and the basic instruments needed to control a vehicle such as a steering wheel, and gas and brake pedals [122,129]. However, most papers relied on medium or high-fidelity simulators. Among the most used, we can mention the CARRS-Q Advanced Driving Simulator (present in the nine studies listed above), the Foerst Driving Simulator located at the National Technical University of Athens (seven studies: [27,63,77,102,103,123,124]), the PatrolSim high-fidelity driving simulator (four studies: [106,119,128,139]), and NADS-1 (present in the three studies cited above). Most systems are developed by Systems Technology Inc., Hawthorne, CA, USA, both hardware and software (encountered in 17 articles) and Realtime Technologies, Inc., Royal Oak, MI, USA (five studies). 

Regarding the type of display, the system using monitors differs from the systems based on screens for projection. In the examined papers, 40 studies used monitors, ranging from a system with one to five monitors, while in 41 studies, the display system was based on projection. The number of screens on which the images were projected ranged from 1 to 16. Two papers did not report clearly the information related to the display. The visual field of view (FOV) varied between 40° to 360° for the horizontal view and between 30° to 45° for the vertical view. However, this information is not reported in many articles (21 publications). The most advanced display is installed on the NADS-1 simulator, composed of sixteen high-definition LED projectors that provide 360 FOV to drivers [116]. 

The environment of the simulated scenarios contains different road conditions (urban/rural/highway, single lane, multilane) with lengths between 1.9 km to 42.7 km. The complex scenarios contain both rural and urban roads as well as rural, urban roads, and motorways, such as in [63,102,140,141]. The authors provided the lengths of the road in either kilometers, meters, miles, or feet. However, in this research, all these units’ measures were converted into kilometers. Thus, the longest route, 42.67 km, is presented in [90]. Only 48 of the papers reported the duration of the experiment, which varies between 1.5 min [79] to 90 min [96].

Eight papers mention that a manual transmission was utilized in the experiments ([52,68,76,89,104,105,142,143]), and fourteen papers reported that the simulator used an automatic transmission ([69,71,80,90,91,92,99,106,107,118,119,132,136,144]). The other studies did not offer data regarding the vehicle’s transmission.

The simulator’s hardware and software systems were utilized to gather data on the driver’s behavior, although, in 8.43% of the studies, additional driver tracking devices were also used. For example, a device for tracking the driver’s gaze was utilized in six articles, and brain–computer interface (BCI) devices were used in one article. Some researchers used conventional video cameras for eye tracking and manually processed the recorded video to extract the information [89]. In contrast, others used advanced eye tracking devices: the Mobile-Eye head-mounted eye tracker developed by Applied Science Laboratories, Inc., Cambridge, MA [97,145,146], the eye tracking system developed by Seeing Machines, Ltd.: faceLAB™ 4.6 [136], and SmartEye6.0 [121]. An Ewave portable electroencephalographic (E.E.G.) device in [130] measures brain activity. 

The physiological data collected from the participants during the experiment were considered in four studies. The heart rate was measured using devices such as the Nellcor Puritan Bennett SRC-2 pulse oximeter [72,73], the MP100 BIOPAC system [147], and heart rate plus other cardiovascular reactivity indicators (root mean square of successive differences, systolic blood pressure, diastolic blood pressure, and mean arterial pressure) [96].

The studies included in the review have been classified into four classes (A, B, C, and D), according to the characteristics of the driving simulator and the criteria proposed in [59]. The results are presented in Table 3. It can be observed that most studies used a B class simulator (51 papers), followed by a D class (13 papers), A class (12 papers), and C class (7 papers).

### 4.4. RQ3: What Measures Were Used to Predict and Analyze Distraction?

**Figure 7:**
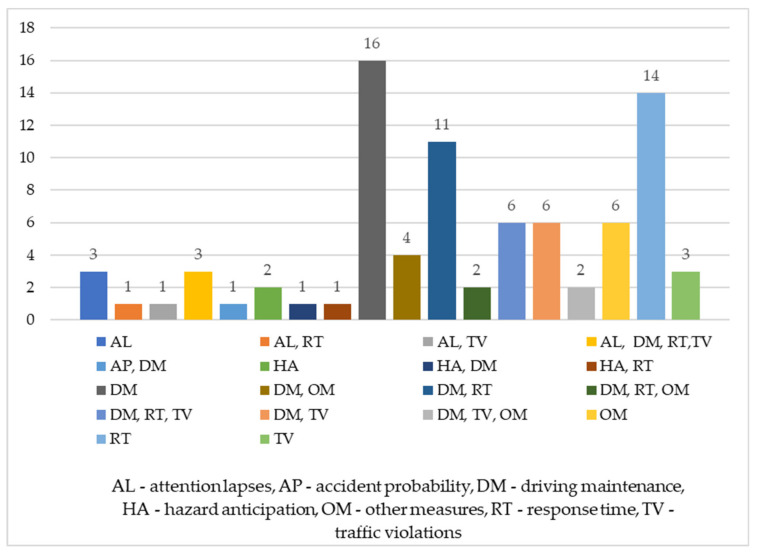
Distribution of papers according to the categories of driving performance measures.

Researchers have proposed several measures that can help evaluate distractions’ effect on the driver’s behavior. Most of them are dependent variables from the driving simulator that are utilized to assess the driving performance while navigating in normal (or baseline) conditions and one or several distracting scenarios. A significant advantage of using a driving simulator is having complete control over the virtual environment and the parameters that can be monitored without additional equipment. Following the classifications from [91,131], the driving performance measures were grouped into seven categories. We have added a new category that encompasses other variables that are not directly related to the vehicle performance parameters: traffic violations (TV), driving maintenance (DM), attention lapses (AL), the response time (RT), hazard anticipation (HA), accident probability (AP), and other measures (OM). The distribution of the papers according to these categories is presented in Figure 7. In some studies, variables belonging to only one category are used, while in others, they are part of two, three, or even all four categories. Most articles used measures from the DM category (50 studies), followed by RT (38 studies), OM (14 studies), TV (21 studies), AL (8 studies), HA (4 studies), and AP (1 study). 

The driving maintenance category includes speed variables such as the mean speed [40,75,104,136], the standard deviation (SD) of speed [40], lane-keeping characterized by the standard deviation of the lateral position (SDLP) [144] or the root mean standard error (RMSE) [118], headway measured in meters—distance headway [100,102]—or in seconds—time headway [92], and the steering control characterized by the steering angle [89,136] and the SD of the steering angle [145].

The response time category mainly refers to the brake reaction time [105], as well as other time variables measuring the response to specific pop-up events such as a sudden appearance of a pedestrian or an animal crossing the road [103]. The traffic violations category includes speeding [91,123], stop signs violations [131], and the number of collisions [108,119]. Attention lapses refer to the outcomes characterizing failures in negotiating an intersection [91] or a roundabout [117]. Hazard anticipation includes various HA variables [82,93]. Lastly, the other measures category includes the variables that fail to meet the criteria of the above-mentioned categories. As such, OM refers to the task completion time [72], the workload [44], and specific eye-tracking variables (the number of glances [142] or off-road glances [120]). The most popular measures assessed in the included articles are presented in Table 4. As can be seen and in line with [101,143], the brake reaction time and standard deviation of lane positioning (SDLP) are the most often reported performance parameters for evaluating the mobile phone use effects.

Besides the measures that characterize the driving performance by means of specialized sensing equipment or self-reported, some studies reported additional parameters regarded as independent variables. Twenty-four articles were found to include information regarding the following independent variables: road configuration (RC), age of participants (A), gender of participants (G), driving experience (E), and traffic flow (T). 

**Figure 8:**
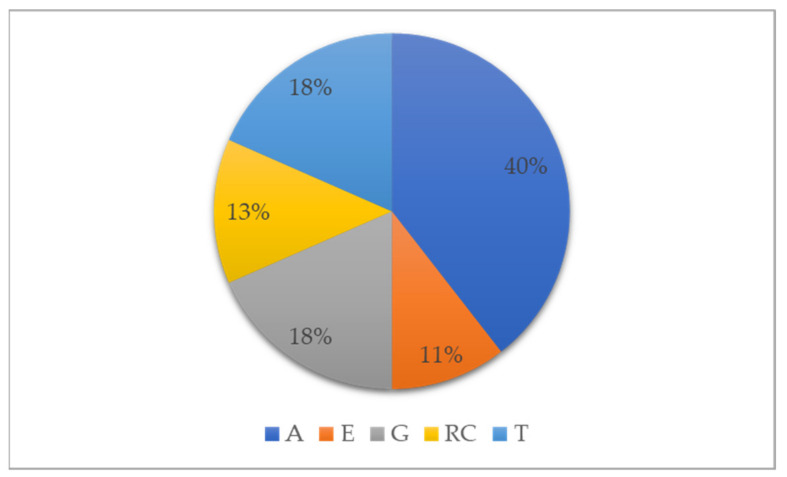
Additional parameters evaluated in the experiments (A—age, E—driving experience, G—gender, RC—road configuration, T—traffic flow).

The distribution of parameters within the 24 articles is shown in Figure 8. In most studies, age was considered as an independent parameter (fifteen studies), as then gender (seven studies), traffic flow (seven studies), road configuration (five studies), and driving experience (four studies). There are studies that consider two or more parameters: A and E [68]; A and G [73,105,116]; A and T [102]; RC and T [104]; A, G, and T [124]; and A, G, E, and T [101,123]. 

The preferred statistical method applied in 48 of the studies was the analysis of variance (ANOVA), followed by the *t*-test with eleven studies, the Wilcoxon signed rank test with seven studies, the Wald test with five studies, and the regression analysis and linear mixed models with three studies each. 

## 5. Discussion

In order to propose relevant collision prevention measures, understanding the impact of mobile phone conversations on drivers’ performance is essential. In this regard, this paper aims to summarize the work that focused on the impact of the use of mobile phones on specific parameters of driving performance among drivers in experiments conducted in car simulators. We found a reasonably large number of studies (n = 83) related to the effects of using a phone in several simulated driving scenarios.

### 5.1. Findings

The findings are consistent with previous simulator-based studies, which reported that TPWD has a negative influence on driving performance. Some observations will be discussed below. 

First of all, it was found that the analyzed studies can be divided into two broad categories depending on the type of distraction, namely some that use phone conversations on hand-held or those who use hands-free devices. Each category introduces distractions, which can be classified into four types: cognitive, visual, manual, and auditory. Of course, a certain secondary task may include more of these distractions which can affect the driving performance. Obviously, the task of driving a vehicle introduces all types of the above-mentioned distractions that the driver’s brain has to manage [107], and additional distractions increase the mental workload and, inherently, the risk of unwanted road events that may occur.

The objective, the number of experiment subjects, the infrastructure employed to pursue the planned objective, and other factors were found to vary throughout the selected articles. The main findings of these investigations do, however, concur. Thus, in connection with talking on the phone while driving, the researchers showed that distracted participants found it more difficult to keep speed variations under control [76], committed more traffic violations or attention lapses [131], and failed to take any action to reduce their speed as they approached some hazards [91]. Cognitive distraction impaired the accumulation of traffic-related information [66,147] and the drivers’ awareness of the safeness of their driving [80] by diverting attention to an engaging cognitive context other than the one associated with driving [65]. It has a negative effect on: latent hazard anticipation [145], reaction time [64,107], headway [93], lane and speed maintenance [111], and time to collision [132], leading to more traffic violations [92]. Even though people recognize these negative effects, they continue to use mobile phones while driving, being deceived by illusory control [129], which leads them to believe that they can control the distraction. Regarding the complexity of phone conversations, studies have shown that the driving performance decreases as the cognitive load involved in the dialogue increases [119,144].

In terms of distraction type, the visual-manual distractions have an impact on the variability of the lateral lane position [44], the average speed [87], since the driver’s eyes are diverted from the road [89], and the mental effort is increased [130]. Although auditory distraction has been studied less, it also seems to affect the drivers’ performance by negatively affecting the brake reaction times [138] and increasing driving infractions [109]. 

Some findings about the independent variables may be drawn from the examined papers. The driver’s age influences their driving performance in various situations. For instance, the use of HF phones significantly affects parameters such as acceleration, lane deviation, reaction time, and accuracy more for older drivers than younger ones [71]. However, young people are prone to more crashes [67], have lower longitudinal control during distracted driving [68], and are more likely to accept a gap in intersections [158]. Middle-age participants drove more slowly than other groups [73] and had more difficulties maintaining their performance and familiarizing themselves with the use of a driving simulator [123]. The age may be counterbalanced by driving experience [123]. In terms of gender, it was found that the male drivers drove at higher speeds [105], but female drivers are more likely to run through the yellow light compared to males [75,116]. 

Road configuration is another parameter that was investigated in the selected studies. Curved roads and vertical alignments have been found to have a stronger impact on the speed and lateral position [99], whereas urban roads have been found to have an impact on speed adaptation behavior [112] and the average reaction time [141]. Moreover, it was found that phone use while driving reduces driver awareness and increases the likelihood of a crash in work-zone areas [146]. Traffic flow has no significant effect on the reaction time and crash probability [63], but in reverse, the engagement in a distraction task would lead to behaviors that can obstruct traffic flow [74]. 

Other findings suggest that HF conversations produce more changes in driving behavior than alcohol for both the longitudinal and lateral driving performance [127], and cognitively demanding HF conversations represent a significant risk to driving compared to the legally permissible blood alcohol concentration [95]. Compared to conversing with an in-car passenger, cell phone conversations increase the number of driving errors [119]. In the first situation, no statistically significant effects were found compared to undistracted driving [103], but even involvement in a conversation with a passenger may decrease the driver’s situation awareness of upcoming hazards [91]. Other activities, such as eating and drinking while driving, have less distracting effects on the driver’s behavior than a phone conversation [25,26]. Studies that have taken physiological data into account have shown that phone conversation distraction increases the heart rate [72,147] and blood pressure [96] compared to driving with no secondary tasks. 

The complexity and duration of the conversation can have a significant impact on driving behavior by increasing the mental workload. As such, a short and simple conversation was found to not influence the driving performance, as opposed to longer and more complex conversations [130,153]. 

In conclusion, studies on distracted driving related to the use of mobile phones in driving simulators have shown that secondary tasks, such as telephone conversations, have different negative effects and directly influence safe driving performance. In the experiments carried out in the analyzed studies, it was shown that the crash probability is increased up to four times by distractions caused by the use of a mobile phone while driving [105].

### 5.2. Recommendations

Qualitative studies can offer novel insights regarding the experiences of distracted driving in simulated conditions that would be otherwise difficult to obtain through a quantitative approach. Open-ended questions and interviews are common qualitative tools that can be used to investigate the user’s perception and awareness on the effects of using a mobile phone while driving. However, most studies are quantitative and use close-ended questions or Likert-type scales to assess the driver’s experience. Nonetheless, one study actually found that participants are not aware of the safeness of their driving when engaged in a cell phone conversation [80]. Moreover, drivers are less able to self-regulate their secondary task because of their diminished self-awareness of the risks posed by cell phone usage. Future research could focus on the driving behavior of young adults and teens, and what factors can predict the risk of engaging in secondary activities, such as texting, calling, browsing social media, and so on.

Drivers experience numerous distractions and their deleterious effects will probably increase if nothing is done. One possible solution is to develop custom simulator training based on the experience of the driver, which could prove to be more relevant as novice drivers can have different needs and desires than experienced drivers. However, age is also another factor to take in consideration when designing an experiment, as age could be a predictor for estimating the difficulty of acclimating to a simulated driving environment.

More research is needed to evaluate the workload, self-perception, multitasking, hazard anticipation, and situational awareness of distracted driving by quantitative and qualitative studies. In addition, there is an increased interest in applying deep learning techniques to analyze and assess the driver’s behavior, as well as to estimate road safety under different driving conditions. Lastly, future studies should aim to discover new connections between the motivation, desire, or contextual factors which influence the driver’s ability to safely operate a vehicle while engaging with a mobile phone.

## 6. Conclusions

This scoping review aimed to offer an overview of the simulator-based studies that addressed talking on a mobile phone while driving. Three research questions were considered that can help to gain new knowledge that could improve future distracted driving studies. The first RQ is focused on the sources of distractions and reveals that cognitive and cognitive–visual distractions are the most important when engaged in talking while driving. The second RQ summarizes the hardware devices used in the studies and has shown that more than three-quarters of the studies were carried out in fixed-based simulators. Although these types of simulators have well-documented results, the main drawback is that participants might underestimate the risk of a crash when involved in secondary activity. Moreover, we have noticed that less than 9% of the studies have used dedicated hardware to monitor the driver’s gaze or other physiological parameters. Therefore, some meaningful insights might have been missed. The third RQ aimed to present the main measures analyzed by researchers to characterize driving behavior. The most used measures were driving maintenance, response time, and a combination of these two. Going further, the most popular variables to describe the driving performance were the reaction time, the mean speed, headway, and the standard deviation of lane position.

We want to emphasize the need for the improved appraisal of crash risk and road safety evaluation in the context of talking on a mobile phone while driving. This is also motivated by the increasing number of road participants and their need to use their mobile phone while driving. Although some drivers adopt compensatory measures, such as a reduced speed and an increased awareness of the surrounding environment, any secondary activity during driving can affect road safety and should be avoided.

The current literature review contributes to the research literature and faces certain limitations which should be mentioned. Many of these limitations are related to the data since incomplete information has been reported in the examined research literature. 

As the use of the telephone while driving is a widely studied research area, some pertinent papers may have been overlooked even after a thorough literature search. Additionally, two other limitations are the language in which the analyzed studies were written and the area in which they were published. The present review examines studies published strictly in English and was restricted to the exclusion of studies published as book chapters or conference proceedings. 

Furthermore, in the present review, methodological limitations, such as the use of small samples, the length of experiments, genders, and others, make it difficult to evaluate the effects of TPWD. 

## Figures and Tables

**Table 1:** Extracted information grouped by categories.

No.	Research Question	Extracted Information
1	Characteristics of studies	first author,
		year of publication,
		journal name,
		country where the experiment took place, institution where the research was conducted
2	What are the main sources of distractions that influence the driver’s behavior?	source of distraction
		distraction task
		scenario type
3	What types of hardware devices were used during experiments to analyze the driver’s behavior?	type of simulator
		motion system
		driving scenario
		tracking devices
		display system
		route length
		experiment duration
4	What measures were used to predict and analyze distraction?	analyzed measures
		independent variables
		statistical analysis technique

**Table 2:** Distribution of papers by research institution and by journals.

Institution	No. of Publications	Journal	No. of Publications
Queensland University of Technology, Australia	8	*Accident Analysis & Prevention*	21
University of Utah, USA	7	*Transportation Research Part F: Traffic Psychology and Behaviour*	12
National Technical University of Athens, Greece	6	*Transportation Research Record*	9
Indian Institute of Technology (I.I.T.), Bombay, India	4	*Traffic Injury Prevention*	5
University of Alabama at Birmingham, USA	4	*Advances in Transportation Studies*	4
Massachusetts Institute of Technology, USA	3	*Human Factors*	4
University of Massachusetts, USA	3	*IATSS Research*	2
Beijing Jiaotong University, China	2	*Journal of Advanced Transportation*	2
Delft University of Technology, The Netherlands	2	*Journal of Safety Research*	2
Israel Institute of Technology Haifa, Israel	2	*Journal of Transportation Safety and Security*	2
University of Minnesota, USA	2	*Perceptual and Motor Skills*	2
University of Roma Tre, Italy	2	*Psychological Science*	2
University Parkway, USA	2	*Psychonomic Bulletin & Review*	2

**Table 3:** Driving simulators classification, according to [59].

Class	Characteristics	Studies
A	*Visual*: basic visual capability, minimum horizontal FoV:40 and vertical FoV:30 *Sound*: engine, rotor, transmission sounds *Motion platform*: no requirement	[26,66,72,83,88,90,114,122,129,142,144,148]
B	*Visual*: system brightness, visual cues, minimum horizontal FoV:80 and vertical FoV:30 *Sound*: cabin sounds *Motion platform*: no requirement	[25,27,52,58,62,63,64,65,67,68,69,70,71,74,77,78,80,82,89,91,92,95,96,97,102,103,104,105,106,109,111,119,120,121,123,124,126,127,130,131,139,140,141,143,145,146,147,149,150,151,152]
C	*Visual*: daylight, night and visual scenes, minimum horizontal FoV:120 and vertical FoV:30 *Sound*: windshield wipers, precipitation, wheels and braking *Motion platform*: yes, with motion cues and special driving effects	[76,98,101,132,133,138,153]
D	*Visual*: advanced scene features (realistic environment), minimum horizontal FoV:180 and vertical FoV:40 *Sound*: realistically acoustic environment *Motion platform*: yes, minimum 6 DoF	[55,93,99,107,112,113,116,118,125,135,136,154,155]

**Table 4:** Main measures used in the experiments of the analyzed studies.

Measure	Units	Description	References
Reaction time	S (or ms)	Time interval between the appearance of an event on the road and the moment when driver starts to brake	[25,27,63,64,65,66,77,79,81,82,90,91,92,95,101,102,103,104,106,107,111,128,131,132,133,138,139,140,141,142,144,150,156]
Number of crashes	counts	The total number of collisions when the driver collided with either another vehicle or object	[26,67,69,74,78,81,82,91,92,106,109,120,126,128,144,157]
Following distance	m	The distance prior to braking between the rear bumper of the pace car and the front bumper of the participant’s car [128]	[26,78,106,119,128,137,139,140]
Deceleration	m/s^2^	The action taken by the driver to avoid a collision	[25,88,118,140,150]
Accident probability	%	An estimated probability for a driver to meet with an accident during sudden events	[63,77,105]
Headway - distance headway	m	The straight-line distance from the center of the driver’s car to the center of the lead car [125]	[82,101,103,125,144]
- time headway	s	Time to the ahead driving vehicle	[93,103,127,130,133,156]
Time-to-collision	s	The time remaining until a collision between the driver’s vehicle and the pace car if the course and speed were maintained [128]	[91,92,101,128,132,149,151]
Speed violation	counts	How many times the vehicle exceeded the speed limit along the route	[69,92,109,124,126],
SD of speed (speed variability)	km/h	The measure of speed variation along the route traveled	[52,55,62,67,71,76,77,89,99,102,111,141]
Mean speed (average speed)	mph (km/h, m/s)	The mean speed of the driver along the route [102]	[25,52,67,68,77,88,89,92,98,99,102,105,116,119,123,133,136]
SD of lane position	m	Variation in distance from center of lane	[55,58,62,67,68,81,95,99,102,103,111,123,127,136,137,143,147]
Heart rate	bpm	Measure of physiological arousal and as an index of the body’s response to physical and cognitive workload [72], physiological index of mental effort	[72,96,147]
Workload	score	An interaction of task and system demands, operator capabilities, training, experience, and effort [111]	[55,72,90,111,153]

Note: s—second, ms—millisecond, m—meter, bpm—beats per minute, mph—miles per hour.

## Data Availability

Not applicable.

## Appendix A

**Table A1:** Summary of selected studies.

ID	Ref.	Year	NP	Age	M	SD	G (M–F)	ST	LSR [km]	TD	MT	Type of Device—Distraction Task
1	[90]	2010	45	NR	20.03	1.58	16–29	fixed-based	42.67	C	DM	HF—conversation
2	[76]	2020	78	NR	23.58	1.55, 1.96, 2.14, 3.36	NR	2 DOF	NR	C	RT	HF—conversation
3	[131]	2006	36	20–53	22.5	NR	NR	fixed-based	8.69	C	TV, DM, AL, RT	HF—conversation
4	[140]	2011	30	24–34	NR	NR	15–15	fixed-based	3.3 + 7.5 + 10.3	C, M	RT	HH, HF, HFV—answering the call
5	[77]	2017	25	NR	62.25	7.2, 7.7	8/5, 7/5	fixed-based	2.1	C	RT, DM	HH—conversation with passenger/at phone
6	[142]	2009	30	18–50	34	11	15–15	fixed-based	18	V, C	AL	HF—answerphone, receive calls
7	[141]	2018	33	23–35	27.5	4.1	17–16	fixed-based	3.3 + 7.5 + 10.3	V, C + RC	RT	HH, HF, HFV—answer a phone call while driving
8	[91]	2009	119	17–59	27.65	10.55	56–61	fixed-based	25.3	C	RT, HA	HF—conversation with in-car passengers, hands-free cell phones, and remote passengers who could see the driver’s current driving situation
9	[143]	2017	100	<30, 30–50, >50	24.14, 36.05, 54.67	2.79, 5.43, 5.04	87–13	fixed-based	3.5	V, C	DM	HH—simple conversation, complex conversation, simple texting, and complex texting tasks
10	[104]	2017	100	<30, 30–50, >51	24.14, 36.05, 54.68	2.79, 5.43, 5.05	87–13	fixed-based	3.5	V, C + RC, T	RT	HH—simple conversation, complex conversation, simple texting, and complex texting tasks
11	[105]	2017b	100	<30, 30–50, >52	24.14, 36.05, 54.69	2.79, 5.43, 5.06	87–13	fixed-based	3.5	V, C + A, G	DM, AP	HH—simple conversation, complex conversation, simple texting, and complex texting tasks
12	[68]	2019	49	NR	22.12, 37.62	2.45, 7.22	22–3, 25–0	fixed-based	3.5	V, C + A, E	DM	HH—simple conversation, complex conversation, simple texting, and complex texting tasks
13	[106]	2008	60	NR	NR	NR	NR	fixed-based	28.9	C + E	DM, RT	HF—conversation
14	[62]	2008	14	18–22	NR	NR		fixed-based	NR	C, M	DM	HH—cell phone conversation, back seat conversation, text message, Ipod manipulation
15	[119]	2008	96	18–49	20	NR	49–47	fixed-based	38.6	C	DM	HF—conversation with passengers in a vehicle and conversation on a cell phone
16	[145]	2019	24	19–31	24.79	2.97		fixed-based	NR	C + RC	HA	HF—a mock cell phone task (HF)
17	[26]	2016	101	18–57	27.8	8.3	68.33	fixed-based	NR	C, V, M	DM	HH—using a hand-held cell-phone, texting, eating
18	[136]	2013	20	18–41	24.4	6.3	12.8	6 DOF	7	V	DM, RT	HF—conversation
19	[120]	2014	24	NR	20.4	1.7		fixed-based	19.3	V, C	TV, DM	HF—conversation in the car and talking on a hands-free cell phone
20	[125]	2003	63	25–66, 8–18	NR	NR	32–31	6 DOF	NR	V, M + A	DM	HH, HF—answer incoming calls, dialing, retrieve a voicemail message from a specific person using either the hand-held or hands-free phone
21	[116]	2016	69	16–7, 18–25, 30–45, 50–60	NR	NR	20–0, 9–9, 9–8, 8–6	13 DOF	NR	V, C + A, G	AL	HH—conversation
22	[118]	2016	32	18–26	21.5	1.99	16–16	6 DOF	NR	C, M	Al	HF, HH—conversation
23	[107]	2014	32	21.47	21.47	2.0	16–16	6 DOF	7	C	RT	HF, HH—conversation
24	[109]	2013	27	18–29	21.04	6.00	12.15	fixed-based	6.09	C, Au	TV, DM	HH—phone ringing
25	[98]	2006	31	18–25, 30–45, 60–75	21, 37, 66	NR	NR	3 DOF	6	C, V, M + A	DM, HA	HF—operating the vehicle entertainment system and conducting a simulated hands-free mobile phone conversation
26	[121]	2015	40	20–52	32.5	NR	11.29	fixed-based	NR	V, C	OM	HH—touching the touch-screen telephone menu to a certain song, talking with laboratory assistant, answering a telephone via Bluetooth headset, and finding the navigation system from Ipad4 compute
27	[78]	2009	60	<17, 18–19, 20–21, >22	NR	NR	13.47	fixed-based	NR	C	DM, TV	HF—cell phone communication
28	[92]	2010	35	20–53	26.67	9.91	6.29	fixed-based	8.7	C	DM, RT, TV, AL	HF—cell phone conversation
29	[69]	2007	49	14–16, 21–52	14.68, 29.0	0.56, 8.94	12–12, 12–13	fixed-based	NR	C + A	TV, AL	HF—cell phone conversation
30	[52]	2015	16	27–59	37.8	10	10.6	fixed-based	42	C, M	DM	HH—phone conversation
31	[89]	2015	20	27–59	37.65	9.75	14.6	fixed-based	10 + 9	V, M, C	DM, OM	HH—conversation, texting, destination entry, following route guidance
32	[97]	2019	48	19.2, 19.5	19.2, 19.5	0.97, 0.93	NR	fixed-based	NR	C, V	HA	HH—cell phone conversation, coin change task
33	[111]	2004	80	18–27	20.61	NR	46.34	fixed-based	4	C	DM, RT	HF—conversation with passenger and conversation on HF phone
34	[95]	2012	20	23–30	26.20	2.58	10.10	fixed-based	NR	C, M	TV, DM, AL, RT	HF—conversation, HF cognitive demanding conversation, texting
35	[132]	2019	37	31–40	34.5	3.1	11–7, 10–9	1 DOF	4	C	DM, RT	HF, HH—conversation
36	[101]	2016	42	30–40	35	3	21.21	1 DOF	5	C + G	RT	HF, HH—conversation
37	[144]	2006	20	21–29	25.9	2.3	NR	fixed-based	NR	C	DM, RT, TV	HF—conversation
38	[71]	2011	48	NR	23.10, 69.21	1.54, 3.05	12–12, 20–4	fixed-based	NR	C	RT, DM	HF—conversation
39	[122]	2011	33	NR	24.3	6.8	17.16	fixed-based	NR	C, V	OM	HF—conversation with a passenger vs. via hands-free phone
40	[138]	2009	16	19–49	26.2	9.1	7, 9	2 DOF	NR	C, M, Au + tactile	RT	HF—simple (scripted demographic and personal questions), complex (mental math and categorization questions)
41	[146]	2007	38	18–59	26.4	NR	20.18	fixed-based	NR	V	RT	HF—phone conversation
42	[149]	2018	64	22–60	33	10	34, 30	6 DOF	NR	V, C	RT	HH—reading, texting, video, social media, gaming, phoning, music
43	[99]	2020	35	18–29	22.9	4.0	22, 13	6 DOF	10	V, M, C + RC	DM	HF, HH—calling, texting vs. road environment
44	[55]	2018	35	18–29	22.9	4.0	22, 13	6 DOF	10	V, M, C	DM, TV	HF—conversation and visual-manual interaction task
45	[135]	2017	32	18–26	21.5	1.99	16, 16	6 DOF	NR	M, C	DM	HF, HH—conversation
46	[112]	2017	32	18–26	21.8	1.9	16.16	6 DOF	NR	M, C + RC	DM	HF, HH—conversation
47	[113]	2019	35	18–29	22.9	4.0	22, 13	6 DOF	NR	V, M, C	OM	HH—ring a doctor and cancel an appointment, text a friend and tell him/her that the participant will be arriving 10 min late, share the doctor’s phone number with a friend, and take a ‘selfie’
48	[123]	2018	95	18–34, 35–54, 55–75	NR	NR	47.48	fixed-based	2.1 + 1.7	C, V + A, G, E, T	DM	HH—conversation with passenger, cell phone use
49	[102]	2017	87	18–34, 35–54, 55–76	NR	NR	NR	fixed-based	2.1 + 1.7	V, C + A, T	DM, RT	HH—conversation with passenger, mobile phone
50	[103]	2019	95	18–34, 35–54, 55–77	28, 47, 64	3.6, 4.8, 6.5	47, 48	fixed-based	2.1 + 1.7	C, V + A, G, E, T	DM, TV, RT	HH—conversation with passenger, mobile phone
51	[124]	2019	95	18–34, 35–55, 55+	NR	NR	47.48	fixed-based	2.1 + 1.7	C, V + A, G, T	DM, TV, RT	HH—conversation with passenger, cell phone use
52	[25]	2019	90	NR	NR	NR	73.17	fixed-based	3.6	C, V	DM, RT, TV	HH—using the mobile phone, drinking and text messaging
53	[63]	2016	140	NR	69.0, 64.5	7.1, 7.9	62–47, 20.11	fixed-based	2.1 + 1.7	C, V + T	RT	HH—conversation with passenger vs mobile phone use
54	[150]	2018	51	21–49	27	5.7	36.15	fixed-based	10.4	C	RT, AL	HH—phone conversation
55	[154]	2020	36	21–54	33.3	8.6	21–15	fixed-based	4.8	V, Au	DM, RT	HF—features presented via a mobile phone mounted near the line of sight
56	[153]	2004	24	18–32	20.4	NR	12.12	fixed-based	NR	C	DM, RT, OM	HF—phone conversation
57	[127]	2008	45	NR	22.3	NR	45–0	3 DOF	NR	C	DM, RT, OM	HF—respond to instructions, HF conversation
58	[66]	2014	53	NR	NR	NR	NR	fixed-based	NR	C	RT	HF—phone conversation
59	[148]	2008	32	17–21	19.0, 19.3	NR	7.9	fixed-based	NR	V, M	DM, TV, RT	HH—manipulating controls of a radio/tape deck and dialing a hand-held cellular phone
60	[83]	2010	60	NR	20.56, 20.65	2.18, 1.89	14–9, 20–15	fixed-based	56.3	C	TV	HF—phone conversation
61	[72]	2006	35	19–23, 51–66	20.67, 56.82	0.91, 4.5	10–8, 7–10	fixed-based	NR	C + A	DM, OM	HF—a simulated cellular telephone conversation (easy task), two paragraphs from the Wechsler Memory Scale (easy task), segments of the Continuous Performance Task (hard task), and Multiple Interference Task (hard task)
62	[64]	2012	42	NR	NR	NR	15–9, 12–6	fixed-based	15.14	C, Au	RT	HF—processing of a single spoken word
63	[93]	2015	32	21.47	21.47	1.98	16.16	6 DOF	7	C	DM	HH, HF conversation
64	[80]	2016	100	18–41	21.8	NR	33–67	fixed-based	8.2	C	TV	HF phone conversation
65	[155]	2021	45	NR	62.8, 24.3	7.2, 4.8	30–0, 11–4	fixed-based	NR	V, P (postural − physic) + A	DM, OM	HH—texting on a smartphone while sitting on a stable or unstable surface
66	[151]	2014	40	NR	20.47	4.76	24, 16	fixed-based	8.04	V, M	DM, RT	HH—use Google Glass or a smartphone-based messaging interface
67	[129]	2010	69	17–22	19.03	0.69	25–44	fixed-based	NR	C	OM	HH—phone conversation
68	[27]	2019	50	20–60	31	NR	32–18	fixed-based	5	C, V, Au + A	DM, RT	HH, HF—conversation
69	[152]	2020	123	18–64	34.46	13.04	62.61	fixed-based	26.4	V, Au	DM, OM	HH—audio warning, flashing display
70	[126]	2016	50	24–54	39.8	8.4	49, 1	fixed-based	36.2	C, M, V	TV, DM, OM	HH—cell phone conversation, text message interaction, emailing interaction
71	[74]	2013	75	16–18, 19–25	17.67, 23.39	1.18, 1.81	11–19, 23–22	fixed-based	38.6	C, M + T	TV, DM	HH—cell phone, texting
72	[114]	2017	32	18–25	20.6	2.1	32–0	6 DOF	13	V	DM, TV	HH—gamified boredom intervention
73	[65]	2001	72	18–30, 18–26	21.3, 20.5	NR	24, 24; 12.12	fixed-based	NR	C	RT	HH, HF—conversation
74	[147]	2021	18	20–51	27.17	7.96	4.14	fixed-based	28	V, C	DM, OM	HF—phone call: visuospatial questions, and conceptual questions
75	[58]	2015	36	NR	28.44	9.26	30.6	fixed-based	NR	C, V, M	DM	HH—conversation, texting
76	[67]	2013	92	<20, 24–30, >65/NR	18, 26.4, NR/26.4, 18.3, 69.8	0.44, 1.92, NR/1.76, 0.74, 4.2	NR	fixed-based	NR	V, C + A	TV, DM, OM	HF—phone conversation—responding to incoming calls, and initiating calls
77	[82]	2009	30	23–50	32.4	6.75	26.4	fixed-based	NR	C	TV, DM, RT	HH—phone conversation
78	[139]	2016	23	18–40	23.26	NR	13.10	fixed-based	17.5	C	DM, RT	HF—phone conversation
79	[96]	2018	60	NR	19.74	2.4	30.3	fixed-based	8.04	C, M	OM	HF—conversation, texting
80	[133]	2020	42	30–40	34.33	2.99	21.21	1 DOF	NR	C	RT	HF, HH—conversation
81	[70]	2020	34	NR	47.6, 23.05	NR	23, 11	fixed-based	NR	V, M + A	OM	HF—normal conversation (non-emotional cellular conversation), and seven-level mathematical calculations
82	[88]	2021	101	18–57	27.8	8.3	68, 33	fixed-based	6	V, C, M	DM	HH—texting, talking on the phone, or eating
83	[130]	2020	34	NR	32.5	5.38	17, 17	fixed-based	NR	C	DM, OM	HF—phone conversation

Note: NP—number of participants; M—mean; SD—standard deviation; G—gender; ST—simulator type; LSR—length of simulated route; MT—measure type; AL—attention lapses; AP—accident probability; DM—driving maintenance; HA—hazard anticipation; RT—response time; TV—traffic violations; OM—other measures; HH—hand-held; HF—hands free; HFV—horizontal field of view.
